# Clinical, genomic, and neurophysiological correlates of lifetime suicide attempts among individuals with alcohol dependence

**DOI:** 10.21203/rs.3.rs-3894892/v1

**Published:** 2024-02-09

**Authors:** Peter Barr, Zoe Neale, Chris Chatzinakos, Jessica Schulman, Niamh Mullins, Jian Zhang, David Chorlian, Chella Kamarajan, Sivan Kinreich, Ashwini Pandey, Gayathri Pandey, Stacey Saenz de Viteri, Laura Acion, Lance Bauer, Kathleen Bucholz, Grace Chan, Danielle Dick, Howard Edenberg, Tatiana Foroud, Alison Goate, Victor Hesselbrock, Emma Johnson, John Kramer, Dongbing Lai, Martin Plawecki, Jessica Salvatore, Leah Wetherill, Arpana Agrawal, Bernice Porjesz, Jacquelyn Meyers

**Affiliations:** SUNY Downstate Health Sciences University; SUNY Downstate Health Sciences University; SUNY Downstate Health Sciences University; Icahn School of Medicine; Icahn School of Medicine; SUNY Downstate Health Sciences University; SUNY Downstate Medical Center; State University of New York Downstate Health Sciences University; Indiana University School of Medicine; University of Connecticut; Washington University School of Medicine; Indiana University; Indiana University School of Medicine; Indiana University School of Medicine; Washington University in St. Louis; Downstate Medical Center; State University of New York (SUNY) Downstate Health Sciences University

## Abstract

Research has identified clinical, genomic, and neurophysiological markers associated with suicide attempts (SA) among individuals with psychiatric illness. However, there is limited research among those with an alcohol use disorder (AUD), despite their disproportionately higher rates of SA. We examined lifetime SA in 4,068 individuals with DSM-IV alcohol dependence from the Collaborative Study on the Genetics of Alcoholism (23% lifetime suicide attempt; 53% female; 17% Admixed African American ancestries; mean age: 38). We 1) conducted a genome-wide association study (GWAS) of SA and performed downstream analyses to determine whether we could identify specific biological pathways of risk, and 2) explored risk in aggregate across other clinical conditions, polygenic scores (PGS) for comorbid psychiatric problems, and neurocognitive functioning between those with AD who have and have not reported a lifetime suicide attempt. The GWAS and downstream analyses did not produce any significant associations. Participants with an AUD who had attempted suicide had greater rates of trauma exposure, major depressive disorder, post-traumatic stress disorder, and other substance use disorders compared to those who had not attempted suicide. Polygenic scores for suicide attempt, depression, and PTSD were associated with reporting a suicide attempt (ORs = 1.22–1.44). Participants who reported a SA also had decreased right hemispheric frontal-parietal theta and decreased interhemispheric temporal-parietal alpha electroencephalogram resting-state coherences relative to those who did not, but differences were small. Overall, individuals with alcohol dependence who report SA appear to experience a variety of severe comorbidities and elevated polygenic risk for SA. Our results demonstrate the need to further investigate suicide attempts in the presence of substance use disorders.

## Introduction

Approximately 2–5% of U.S. adults report having attempted suicide in their lifetimes [1–3], with the prevalence increasing in more recent birth cohorts [4]. Additionally, deaths by suicide are one of the leading causes in the recent decline in U.S. life expectancy, alongside other “deaths of despair” such as drug and alcohol related deaths [5, 6]. While the rate of suicide attempts in the general population is alarming, the rate of lifetime suicide attempts is greater than triple (17.5%) among those with an alcohol use disorder (AUD) [7]. Among those seeking treatment for AUD, 40% report at least one suicide attempt at some point in their lives [8–11]. A history of past suicide attempts is among the most prominent predictors of subsequent suicide death and contributes significant health care and disability costs per attempt [12]. Research focused on correlates of suicide attempts can potentially help identify and treat those with non-fatal suicide attempts, with the goal of reducing suicide deaths and saving lives [13]. Individuals with AUD have emerged from these data as a particularly high-risk group.

Genome-wide association studies (GWAS) have identified numerous genomic markers associated with AUD and similar phenotypes [14–17]. For AUD, a recent GWAS from the Million Veteran Program (MVP) [15] identified three loci previously associated with alcohol dependence [16] —*ADH1B, ADH1C*, and *ADH4*, and seven novel loci -- *GCKR, SIX3, SLC39A8, DRD2*, an intergenic variant on chr10q25.1 (rs7906104), and *FTO*. A meta-analysis of AUD between MVP, PGC and the Collaborative Study on the Genetics of Alcoholism (COGA), which included 48,545 AUD cases and 187,065 controls, identified 10 genome-wide significant loci. In terms of AUD-adjacent phenotypes, Sanchez-Roige et al. (2019) meta-analyzed GWAS of the alcohol use disorders identification test (AUDIT) in 141,932 individuals from the UK Biobank and 23andme [14]; replicating previously identified signals in the genes *ADH1B, ADH1C, KLB*, and *GCKR* and finding novel associations localized to genes including *JCAD* and *SLC39A13*. Zhou et al [17] identified 110 independent risk variants in a GWAS of “problematic alcohol use,” meta-analyzing results from MVP, UK Biobank, FinnGen, PGC, and others.

Recent GWASs have also identified genomic markers associated with suicide attempts (SA) broadly [18, 19] and among individuals with psychiatric illness [20, 21]. The Suicide Working Group of the Psychiatric Genomics Consortium, or PGC (formerly the International Suicide Genetics Consortium) recently identified 12 loci in a large scale meta-analysis of 43,871 individual who had a lifetime suicide attempt and 915,025 controls. Importantly, in prior analyses, the top loci SNP on chromosome 7 remained significant after conditioning on GWAS results for depression [20]. Other GWASs have identified genomewide significant variants for SA within those with other psychiatric disorders: rs45593736 was associated with suicide attempt in major depressive disorder, chr4_23273116_D was associated with SA in bipolar disorder, and rs138689899 was associated in the meta-analysis of suicide attempt in mood disorders (bipolar disorder + major depressive disorder). Levey et al [21] found one genome-wide significant signal near *LDHB* (rs1677091) in individuals of European ancestries and three associations among individuals of African ancestries, including: rs683813 (*ARNTL2*), rs72740082 (*FAH*), and rs11876255. Variants within *LDHB* and *FAH* replicated in an independent sample. However, despite the higher rates of suicide attempts, no prior GWAS has examined suicide attempt in the presence of AUD.

In a similar manner to the genetics of suicide and AUD, two separate literatures have explored neurocognitive differences between (a) individuals who have attempted suicide to those who have not [22–24] and (b) individuals with AUD [25–27] compared to those unaffected with AUD. Among those with AUD, deficits in many domains of brain functioning have been observed, including neuropsychological performance, and neurophysiological indices [25–27]. Executive functioning is the primary focus of such studies, with a large literature demonstrating that individuals with AUD display poorer executive functioning and atypical neurophysiological profiles (e.g., EEG connectivity) than individuals without AUD [28–31]. These areas of brain functioning have also been examined among individuals who have exhibited suicidal ideation and related mental health problems (depression) [22–24], though research focused on SA is limited.

While no previous studies have examined EEG connectivity and SA, Leuchter et al. [32] examined EEG connectivity in depressed patients and found evidence of higher alpha and theta coherences in frontal, temporal, and parietal regions, and higher beta coherence in frontal and temporal regions. Further, a recent study found other neurophysiological differences associated with binge drinking and suicidal behaviors in Mexican American and American Indian adolescents [33]. To our knowledge, no prior study has examined neural connectivity among those with AUD who have attempted suicide.

Given the higher rates of SA observed among those with AUD, we explored whether there are clinical, genomic, and neurophysiological markers of SA within this population. Among participants diagnosed with DSM-IV Alcohol Dependence (AD) drawn from the Collaborative Study on the Genetics of Alcoholism (COGA), we first conducted a genome-wide association study (GWAS) of SA and performed downstream analyses to determine whether we could identify specific biological pathways of risk. Next, to explore risk in aggregate, we examined whether clinical risk factors, polygenic scores (PGS) for comorbid psychiatric problems, and neurocognitive functioning differed between those with AD who have and have not reported a lifetime suicide attempt.

## Methods

### Sample and Measures

The Collaborative Study on the Genetics of Alcoholism (COGA) is a large, multi-site study of 17,854 participants from 2,255 families affected with AUD, designed to identify and understand genetic factors involved in the predisposition to alcoholism and related disorders, as previously described [34–36]. Participants 18 or older completed the Semi-Structured Assessment for the Genetics of Alcoholism (SSAGA) which is a poly-diagnostic interview [34], and participants ages 12–17 completed an adolescent SSAGA. All participants were queried about whether they had “ever tried to kill” themselves (*suicide attempt*), regardless of a history of suicidal ideation (i.e., thoughts about killing yourself). Importantly, suicide attempt items were not exclusively nested within the diagnostic section for major depressive disorder (MDD), although individuals who reported suicide attempts in that section were coded accordingly as having reported the behavior.

Suicide attempt (SA) data derived from the SSAGA was available on 4,068 COGA participants with an alcohol dependence diagnosis (lifetime) and GWAS data (including 3,270 individuals of European ancestries and 798 individuals of African ancestries). For the current analyses, we included individuals reporting any suicide attempt, including those reporting drug-related suicide attempt (14% of all attempts). We created diagnoses of alcohol dependence, other substance dependence, other psychiatric disorders, suicidal thoughts and behaviors, and trauma exposure based upon DSM-IV criteria using the child and adult versions of the SSAGA. We assessed nicotine dependence using the Fagerström Test for Nicotine Dependence (FTND) scores [35]. Additionally, we included measures of sociodemographic characteristics, extended family histories of AD, and other alcohol-related problems (see supplementary information for a full description).

### GWAS data

Genotyping, imputation and quality control have been described previously [36]. Briefly, genetic data were used to assign ancestry and families were classified as primarily European (EUR) or Admixed African American (AFR) ancestries according to the ancestry of the greatest proportion of family members [36]. Genotyping of 798 AFR individuals and 3270 EUR individuals included in the analytic sample was performed using the Illumina 2.5M array (Illumina, San Diego, CA, USA), the Illumina OmniExpress [37], the Illumina 1M array, or the Affymetrix Smokescreen array [38]. SNPs with a genotyping rate <98%, Hardy-Weinberg equilibrium violations (p<10^−6^), or with minor allele frequency (MAF) less than 3% were excluded from analyses. Mendelian inconsistencies were removed, after which data were imputed to 1000 genomes (Phase 3) using SHAPEIT [39] and IMPUTE2 [40]. Following imputation, dosage probabilities ≥ 0.90 were converted to hard calls. Mendelian errors in the imputed SNPs were reviewed and resolved as described previously [41, 42]. SNPs with an imputation information score < 0.30 or MAF < 0.03 were excluded from subsequent analysis.

### Polygenic scores (PGS)

We estimated polygenic scores (PGS), which are aggregate measures of the number of risk alleles individuals carry weighted by effect sizes from GWAS summary statistics, for a variety of psychiatric and substance use phenotypes. We included PGS derived from recent GWAS of (1) alcohol use disorders (AUD) [43], (2) depression (DEP, 23andMe excluded) [44], (3) post-traumatic stress disorder (PTSD) [45], (4) bipolar disorders (BIP) [46, 47], (5) schizophrenia (SCZ) [47, 48] (6) smoking initiation (SMOK, as a proxy for externalizing risk) [49, 50] and (7) suicide attempt (SUI) [19]. For AUD and BIP, we meta-analyzed published GWAS results with corresponding results from FinnGen (release 9, see supplemental information for results) [51]. We focus on these PGS specifically because: 1) these disorders are phenotypically correlated with suicide attempt, and 2) they contain GWAS results for both European and African ancestries. For GWAS that originally included COGA in the discovery sample, we obtained summary statistics with COGA removed.

To date, GWAS have been overwhelmingly limited to individuals of European ancestries [52]. Because of variation in allele frequencies and linkage disequilibrium (LD) patterns, PGS often lose predictive accuracy when there is mismatch between the ancestries of the discovery GWAS and target sample [53, 54]. COGA includes participants of both African and European ancestries, thus we used PRS-CSx [55], a method that integrates GWAS summary statistics from well-powered GWAS (typically of European ancestries) with those from other populations to improve the predictive power of PGS in the participants of African ancestries in COGA. PRS-CSx employs a Bayesian approach to correct GWAS summary statistics for the non-independence of SNPs in LD. We converted PGS into Z-scores for ease of interpretation

### Electroencephalogram (EEG) data

EEG recording and processing have been detailed previously [56]. Briefly, resting (eyes-closed) EEG was recorded for 4.25 min; a continuous interval of 256 seconds was analyzed. Each subject wore a fitted electrode cap using the 61-channel montage as specified according to the extended 10–20 International system. The nose served as reference and the ground electrode was placed on the forehead. Electrode impedances were always maintained below 5 kΩ. EEG was recorded with subjects seated comfortably in a dimly lit sound-attenuated temperature-regulated booth. They were instructed to keep their eyes closed and remain relaxed, but not to fall asleep. Electrical activity was amplified 10,000 times by Neuroscan and Masscomp amplifiers, with a bandpass between 0.02 Hz to 100 Hz and recorded using the Neuroscan system (Compumedics Limited; El Paso, TX). EEG procedures were identical at all COGA collection sites. Bipolar electrode pairs were derived to reduce volume conduction effects, and 27 representative coherence pairs were selected based on previous EEG coherence work in COGA [56]. Magnitude squared coherence was calculated from power spectral values derived from Fourier Conventional Fourier transform methods [57]. Coherence measures were generated between bipolar pairs at the following frequency bands: theta (3–7 Hz), alpha (7–12 Hz), beta (12–28 Hz).

### Statistical analyses

We compared those with AD who reported a suicide attempt and those with AD who did not report a suicide attempt across a range of sociodemographic, clinical, and other measures. Multiple-group, multi-level regression models were conducted in Mplus [58] and adjusted for sex, age (at time of psychiatric assessment), ancestry, family history of AD, and family relatedness. We ran all models simultaneously (i.e., correlation among all variables accounted for) accounting for multiple testing.

We conducted GWAS, on 7,784,968 SNPs in the EUR sample and 16,100,604 SNPs in the AFR sample using a mixed model incorporating a genetic relationship matrix to control for relatedness [59] in the *GWAF* package in R [60]. We included sex, age, the first three ancestral PCs (PC1-PC3), genotype array, and birth cohort (prior to 1930, 1930–1949, 1950–1969, and 1970 and after) as covariates. GWAS were stratified by ancestries, using identical phenotypic definitions, covariates, SNP QC standards, MAF thresholds and imputation protocols. Subsequently, we meta-analyzed across the AFR and EUR results using inverse-variance fixed-effects weighting and genomic control in METAL [61]. We used established thresholds for genome wide significance (p< 5 × 10^−8^). We also conducted a post-hoc GWAS analysis covarying for depression, given the high prevalence.

Next we performed a series of post-GWAS analyses using a protocol outlined in previous analyses [62]. We limited results to the EUR only given the small sample size of the AFR analyses and the lack of AFR predicted transcriptomic expression results in some of the post-GWAS pipelines. To identify functional enrichment, we used MAGMA software (version 1.08), and its recent intersessions (FUMA version 1.3.6) [63], a method for gene-level and gene-set enrichment analysis using GWAS summary statistics. In all the MAGMA-based analyses, SNPs were annotated to the 20,260 coding genes from Ensembl v92, with a 1 kb window for both sides (i.e., start and end). Since GWAS contained EUR and AFR samples, we used the 1000G European and African panels [64] respectively to account for linkage disequilibrium (LD) between SNPs. Finally, we corrected all tests for multiple-testing using a Bonferroni correction.

Next, we used the summary-data-based Mendelian randomization (SMR) method to test for a joint association between GWAS summary statistics SNPs and eQTL, using the default settings in the SMR software [65] and the 1000G European ancestries reference panel. We again applied a Bonferroni correction for multiple-testing on the SMR P-value (PSMR). Moreover, a post-filtering step was applied by conducting heterogeneity in dependent instruments (HEIDI) test. The HEIDI test distinguishes the causality and pleiotropy models from the linkage model by considering the pattern of associations using all the SNPs that are significantly associated with gene expression in the cis-eQTL region. The null hypothesis is that a single variant is associated with both trait and gene expression, while the alternative hypothesis is that trait and gene expression are associated with two distinct variants. We defined significant hits based on SMR-HEIDΙ as those for which PSMR met the Bonferroni significance threshold and had PHEIDI>0.05.

Lastly, we used the JEPEGMIX2-P software [66] with default settings to conduct TWAS using only the 13 brain-specific GReX models coming from GTeX v8 [67]. This method was preferable since it relied on a covariance matrix based on 33K samples compared to other TWAS methods which use less than 3k samples. We applied the within-tissue Bonferroni correction to detect significant TWAS genes.

For polygenic scores, we first compared those with AD who had reported a suicide attempt to those with AD who had not reported a suicide attempt across all PGSs, independently, using logistic regression in R (version 4.2.1). Second, to ensure that results within those with AD were not biased by conditioning on AD [68], we also compared: 1) those with AD who had a reported suicide attempt, 2) those with AD who had not reported a suicide attempt, and 3) those without AD who had a reported suicide attempt to those who neither reported a suicide attempt nor meet criteria for AD (see Supplemental Table 1 for sample description) using a multinomial logistic regression model in the *nnet* package in R [69]. In both analyses, we included sex, age, the first three ancestral PCs (PC1-PC3), genotype array, and birth cohort as covariates. To adjust for familial clustering, we used cluster robust standard errors [70, 71]. We stratified analyses by ancestry and then meta-analyzed results (by PGS) within each of the analyses above. All analyses were corrected for multiple testing.

Lastly, we compared those with AD who reported a suicide attempt and those with AD who did not report a suicide attempt across neurophysiological measures (resting state EEG coherence) using multiple-group, multi-level regression models were in Mplus. We included covariates for sex, age (at time of assessment), ancestry, family history of AD, and family relatedness.

## Results

### GWAS and Post-GWAS analyses

Within the analytic sample we performed a GWAS of SA in those with available genetic data (no SA = 2,495 EUR and 643 AFR; SA = 775 EUR and 155 AFR). There was no individual SNP associated with suicide attempts that reached genome-wide significance (see supplemental information for full results). For the post-GWAS analyses, there were no significant gene-based or gene-set enrichment from the MAGAMA results (see supplemental tables S1-S2). Additionally, none of the results from the SMR analyses reached significance after correcting for multiple testing (see supplemental tables S3-S5).

### Clinical Risk Factors Associated with Suicide Attempt in Participants with AD

The main analytic sample was limited to the 4,068 participants with a DSM-IV diagnoses of alcohol dependence (AD). We compared 3,138 COGA participants who met criteria for DSM-IV AD and did not attempt suicide in their lifetime with 930 participants with AD who attempted suicide. Overall, those with AD who attempted suicide were more likely to be female (53% vs. 32%). Rates of suicide attempt and the age distribution of participants were similar across ancestry groups (see Table S6 for ancestry stratified results). Table 1 presents the full set of comparisons across groups. The majority (58.4%) of the analytic sample endorsed suicidal ideation at some point in their lifetime; of those who attempted suicide, 97.6% endorsed prior suicidal ideation compared to 46.8% of those who did not attempt suicide. Participants with AD who had attempted suicide were more likely to have been exposed to traumatic events in their life, and to meet lifetime criteria for major depressive disorder, post-traumatic stress disorder, and other drug use disorders compared to those who had not attempted suicide. In addition, participants with AD that reported attempting suicide had higher family history densities of AD [72], started drinking at an earlier age, and had more severe indicators of alcohol-related problems.

### Polygenic Scores

**Figure 1:**
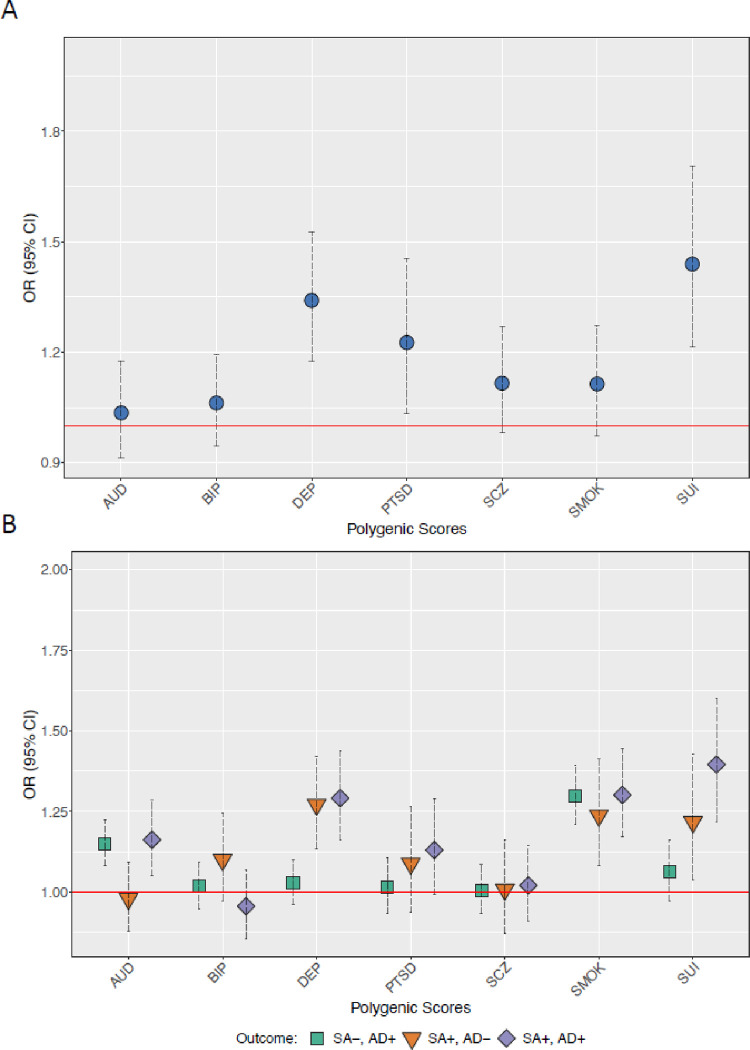
Polygenic Scores for AUD, DEP and SUI across those who have and have not reported a suicide attempt Panel A presents odds ratios (OR) for AUD, DEP, and SUI PGSs from logistic regression models in persons with AD who had and had not attempted suicide. Panel B presents OR from multinomial logistic models (no AD, no suicide attempt as reference group). All models include cohort, sex, PC1-PC3, array, and site as covariates. SEs adjusted for familial clustering using cluster-robust standard errors. AFR = African Ancestries, EUR = European Ancestries, AUD = alcohol use disorder polygenic score, DEP = depression polygenic score, = SUI suicide attempt polygenic score, SA− = no lifetime suicide attempt, AD− = does not meet criteria for alcohol dependence, SA+ = lifetime suicide attempt, AD+ = meets criteria for alcohol dependence.

Figure 1, Panel A presents the meta-analyzed results for associations between each of the corresponding PGSs and lifetime suicide attempt within those meeting criteria for AD. PGSs for DEP (OR_*META*_ = 1.34, 95% CI = 1.18, 1.53), PTSD (OR_*META*_ = 1.23, 95% CI = 1.03, 1.45), and SUI (OR_*META*_ = 1.44, 95% CI = 1.22, 1.70) were associated with increased odds of reporting suicide attempt. However, the AUD, BIP, SCZ, and SMOK PGSs were not associated with suicide attempt (ancestry-specific results in Table S8).

Figure 1 (Panel B) shows conditional PGS results from the multinomial logistic models comparing those with AD who had attempted suicide (AD+, SA+), those with AD who had not attempted suicide (AD+, SA−), and those without AD who had attempted suicide (AD−, SA+) to those without an AD diagnosis and who had not attempted suicide (full results in Table S9. The AUD (OR_*META*_ = 1.16, 95% CI = 1.05, 1.28), DEP (OR_*META*_ = 1.29, 95% CI = 1.16, 1.44), SMOK (OR_*META*_ = 1.30, 95% CI = 1.17, 1.44), and SUI (OR_*META*_ = 1.40, 95% CI = 1.22, 1.60) PGSs were all associated with increased odds of being in the AD+, SA + group. Interestingly, the only differences in results between the AD+, SA + group and the AD+, SA− group was in the DEP and SUI PGSs, while the only differences in results between the AD+, SA + group and the AD−, SA+ group was in the AUD PGS.

### Neurophysiological Findings

We observed nominal differences in resting state EEG coherence patterns of alcohol dependent individuals who had attempted suicide compared to those who had not attempted suicide. However, only two findings withstood multiple test correction: decreased right hemispheric frontal-parietal theta (3–7Hz @ F8-F4-P8-P4) and decreased interhemispheric temporal-parietal alpha (7–12 Hz @ T8-P8-T7-P7) EEG resting-state coherences (p < 0.001, Supplemental Fig. 3). Exploratory analyses within a subset of individuals who had available neurocognitive measures is available in the supplementary information.

## Discussion

Researchers have begun to identify clinical, genomic, and neurophysiological correlates of suicide attempts among individuals with and without psychiatric illnesses (i.e., schizophrenia, bipolar disorder, depression) [13, 14, 15, 16]. However, this has yet to be examined among those with AUD, despite the higher rates of suicide attempts observed among those with AUD. The current study identified distinct clinical, genomic, and neurophysiological associations with lifetime suicide attempt among individuals who meet criteria for alcohol dependence.

In terms of genomic findings, none of the GWAS results or downstream analyses produced any robust associations. Given the large sample sizes necessary for discovery [73, 74] of genetic associations, these results are not surprising. Future studies with larger samples and even greater ancestral diversity are needed to identify genes and biological pathways implicated in SA.

When we examined risk factors in aggregate, participants with AD in COGA had elevated levels of suicidal ideation, other substance use disorders, and trauma exposure compared to the general population [2, 71]. However, among those who met criteria for AD and reported suicide attempts had even greater levels of all types of traumas (sexual, assaultive, and non-assaultive), other substance use disorders, suicidal ideation, and comorbid psychiatric conditions (PTSD and major depressive disorder) relative to those who had not attempted suicide. These results confirm that those with AD who report a lifetime suicide attempt are clinically high-risk group. Future work should utilize prospective information to determine whether these individuals have similar trajectories of psychiatric problems across time.

The polygenic scores for suicide attempt, depression, and PTSD were associated with a lifetime suicide attempt in persons with AD in the meta-analyzed results. Exploration of the ancestry-specific result demonstrate these were primarily driven by the associations in the EUR participants. The lack of associations of PGSs in those of African ancestries likely stems from the relatively small sample sizes of the discovery GWASs [73]. Importantly, in the multinomial logistic regression models, those with AD did not differ in mean levels of AUD PGS regardless of whether they had reported a lifetime suicide attempt. Similar to the logistic regression models limited to persons with AD, those who had attempted suicide had higher suicide and depression PGSs.

We also observed significant decreases in right hemispheric frontal-parietal theta (3–7 Hz @ F8-F4-P8-P4) and interhemispheric temporal-parietal alpha (7–12 Hz @ T8-P8-T7-P7) EEG resting-state coherences in the resting state among AD individuals who had attempted suicide. While there are no prior studies of suicide attempt that examined EEG coherence, differences in alpha and theta coherences in frontal, temporal, and parietal regions, and higher beta coherence in frontal and temporal regions were found previously in depressed patients [76]. Together, these data suggest that while both decreased theta and alpha resting state connectivity are likely among AD individuals with depression and suicide attempts, but more data are needed to make definitive any conclusions.

We note several important limitations. We note that suicide rates have changed significantly over the past several decades, partially spanning the interval of data collection. Additional research is needed in the area of suicide attempts among individuals with substance use disorders and other psychiatric comorbidities. While this study focused on AD, there is also a high rate of suicide attempts among individuals with other substance use disorders, particularly cocaine and opioid use disorders. Additionally, we do not have data on those who died by suicide, which may differ from those who have attempted but not taken their own lives. Lastly, larger and more diverse samples are needed so that the benefits of this research can benefit all segments of the population [77].

Research is beginning to identify risk factors for suicidal behaviors. In the current analysis, we demonstrated that polygenic scores for suicide attempt, depression, and PTSD, and lower neurophysiological functioning were associated with suicide attempts among individuals with AD. Future work with larger and more diverse samples can examine additional risk factors, such as social and environmental conditions. Identifying robust predictive markers within an already high risk group may allow for earlier intervention and prevention from unnecessary loss of human life.

## Figures and Tables

**Table 1:** Sociodemographic Indicators, Trauma Exposure, Psychiatric and Substance Use Disorders in COGA Participants with Alcohol Dependence (N = 4,068)

	No Suicide Attempt (N = 3,138)	Suicide Attempt (N = 930)
*Socio-demographics*		
Female (%)	31.8%	53.1%*
Black or African American (%)	20.5%	16.7%
Hispanic (%)	6.2%	8.5%
Mean age at last interview (SD)	39.9 (11.9)	38.2 (10.5)
*Suicide related behavior*		
Suicidal Ideation (%)	46.8%	97.6%*
*Alcohol Use Disorder Indicators*		
Maximum # of AD criteria endorsed	5.1 (1.7)	5.6 (1.6)*
Maximum # drinks consumed/24hrs	28.8 (18.6)	34.2 (22.1)*
Mean age of AD onset (SD)	24.1 (8.4)	22.5 (7.3)*
Mean age of first whole drink (SD)	15.0 (2.3)	13.7 (2.4)*
Ratio of first-degree relatives with AD	0.4	0.5*
*Trauma Exposure*		
Sexual Assaultive Trauma (%)	23.0%	45.1*
Assaultive Trauma (%)	34.5%	53.8*
Non-Assaultive Trauma (%)	59.3%	71.2*
*DSM-IV Psychiatric Comorbidities*		
Major Depression (%)	9.6%	56.0%*
Panic disorder (%)	2.5%	5.3%
Obsessive Compulsive Disorder (%)	0.7%	5.3%
Social phobia (%)	5.3%	10.6%
Agoraphobia (%)	3.5%	10.8%
Post-Traumatic Stress Disorder (%)	6.3%	19.1%*
Anorexia Nervosa (%)	0.0%	2.1%
Bulimia (%)	3.4%	15.4%
Mania (%)	0.5%	2.1%
Attention-Deficit Hyperactive Disorder (%)	7.1 %	4.3%
Conduct Disorder (%)	31.3%	31.9%
Antisocial Personality Disorder (%)	22.6%	30.9%
Nicotine Dependence (%)	53.8%	73.7%*
Cannabis Dependence (%)	34.7%	28.7%
Cocaine Dependence (%)	29.2%	35.1%*
Stimulant Dependence (%)	13.4%	18.1%*
Sedative Dependence (%)	5.3%	13.7%*
Opioid Dependence (%)	10.4%	12.6%

* *p* < .05
